# Ventricular Tachycardia in Patients With Peripartum Cardiomyopathy: Prevalence, Predictors, and Associated In-Hospital Adverse Events

**DOI:** 10.7759/cureus.56386

**Published:** 2024-03-18

**Authors:** Omar Elkattawy, Ahmed Sabra, Sanjna Patel, Sherif Elkattawy, Julia Delorenzo, Navina Kumar, Mariam Abdeen, Hassan Elsamna, Fayez Shamoon

**Affiliations:** 1 Internal Medicine, Rutgers University, New Jersey Medical School, Newark, USA; 2 Cardiology, Saint Joseph's University Medical Center, Paterson, USA; 3 Internal Medicine, Rowan University School of Osteopathic Medicine, Stratford, USA; 4 College of Medicine, Rutgers University, New Brunswick, USA

**Keywords:** diagnosis of peripartum cardiomyopathy, cardio-obstetrics, peripartum cardiomypathy, ventricular tachycardia (vt), ventricular arrhythmia

## Abstract

Introduction

The purpose of this study was to determine the prevalence of ventricular tachycardia (VT) among patients admitted with peripartum cardiomyopathy (PPCM) as well as to analyze the independent association of VT with in-hospital outcomes among PPCM patients.

Methods

Data were obtained from the National Inpatient Sample from January 2016 to December 2019. We assessed predictors of VT in patients admitted with PPCM. We also assessed the independent association of VT with clinical outcomes among patients admitted with PPCM.

Results

From 2016 to 2019, 4730 patients with PPCM were reported to the national inpatient sample database, 309 of which developed VT (6.5%). Using multivariate analysis, we found predictors of VT to include patient characteristics and factors such as age (adjusted OR (aOR)=1.020, p=0.023), chronic kidney disease (aOR=1.440, p=0.048), coagulopathy (aOR=1.964, p=0.006), and atrial fibrillation (aOR=3.965, p<0.001). Conversely, pre-eclampsia was significantly associated with a decreased risk of VT in PPCM patients (aOR=0.218, p=0.001).

Conclusion

In a large cohort of patients admitted with peripartum cardiomyopathy, we found the prevalence of VT to be 6.5%. Risk factors for VT in this patient population included conditions such as coagulopathy and atrial fibrillation.

## Introduction

Peripartum cardiomyopathy (PPCM) is a rare and understudied cause of cardiomyopathy that occurs during late pregnancy or in the early postpartum period. According to the 2019 Heart Failure Association of the European Society of Cardiology Working Group, PPCM is an “idiopathic cardiomyopathy presenting with heart failure secondary to left ventricular systolic dysfunction towards the end of pregnancy or in the months following delivery, where no other cause is found [1].” The left ventricular ejection fraction in PPCM is almost always less than 45%, and the left ventricle may or may not be dilated [2]. It is marked by a swift clinical progression and a favorable chance for natural recovery but carries a heightened risk of recurrence in future pregnancies [3]. While several probable mechanisms have been proposed for the pathophysiology of PPCM, including viral myocarditis, nutritional deficiencies, autoimmunity, hemodynamic stresses, vascular dysfunction, hormonal insults, and genetic predisposition, there has yet to be any clear consensus in this area [4,5]. Worldwide, peripartum cardiomyopathy affects approximately one in 1,000 pregnancies, with geographic hot spots found in Africa (up to one per 100 pregnancies) and Haiti (one in 300 births) and an incidence of approximately one in 2,230 births in the United States [6]. While the etiology behind PPCM remains unclear, some risk factors for PPCM have been identified, including African descent, pregnancy-related hypertension disorders, multiparity, multiple gestations, obesity, chronic hypertension, and chronic use of tocolytics [4,5].

The topic of pregnancy-related cardiovascular issues has garnered heightened attention recently due to findings indicating their significant role in the rising maternal mortality rates in the United States [7]. Notably, cardiovascular diseases, such as peripartum cardiomyopathy, accounted for 26% of all pregnancy-related fatalities in the United States between 2011 and 2013 [8]. Sudden cardiac death (SCD) often arises following a previous ventricular tachyarrhythmia (VT) episode and accounts for 25-39% of all-cause mortality in PPCM, suggesting a notable presence of VT in this demographic [9]. In an analysis of 9,841 hospital admissions for PPCM in the USA, arrhythmias were found in 18.7% of cases [10]. Among these instances, ventricular tachycardia (VT) was the most prevalent arrhythmia at 4.2%, followed by atrial fibrillation (Afib) (1.3%) and ventricular fibrillation (1%) [10]. A retrospective review by Goland et al. estimated that one in four women with PPCM in the USA experience cardiac arrest secondary to ventricular tachyarrhythmia, thus underlining the need for more research in this area [11]. A better understanding of the pathophysiology and risk factors for mortality in PPCM can expedite the diagnosis and clinical management of PPCM, thereby improving treatment and patient outcomes. This study aims to study the topic of ventricular tachycardia in patients with PPCM, including the risk factors, prevalence, predictors, and associated in-hospital adverse events.

## Materials and methods

Data acquisition 

This is a retrospective study of the National Inpatient Sample (NIS) database. The NIS is part of the Healthcare Cost and Utilization Project (HCUP) set forth by the Agency for Healthcare Research and Quality. It utilizes the International Classification of Disease, Tenth Edition, Clinical Modification (ICD-10-CM) codes for diagnosis and procedures. The data set was utilized to examine the data of patients admitted between the years 2016 and 2019. Encounters with a primary diagnosis of peripartum cardiomyopathy were selected using the ICD-10 code O90.3. This cohort of patients was further divided into patients who developed ventricular tachycardia using ICD-10 code I47.2 during their hospital stay versus patients without this complication. Adult patients ≥18 years old were included. IRB approval was not required as NIS provides de-identified information on patients.

Outcomes and variables

Patient baseline characteristics, such as age, race, household income, and insurance status, were extracted. Comorbidities, hospital complications, mortality rates, disposition status, length of stay, and total charges were also analyzed. 

The primary aim of the study was to assess the characteristics and comorbidities that predict the occurrence of VT in our patient population. We also analyzed the independent association of VT with outcomes taking into account confounders such as age, race, and comorbidities. 

Statistical analysis 

Categorical values were analyzed via Pearson chi-square analysis, and continuous variables were analyzed via an independent Student’s t-test. Logistic regression was performed to generate odds ratios with 95% confidence intervals (CIs) to assess predictors of VT in women with peripartum cardiomyopathy. We also used logistic regression to assess the independent association of VT with outcomes taking into account confounders such as age, race, and comorbidities. A P-value of <0.05 was considered statistically significant. All analyses were completed using IBM SPSS Statistics for Windows, Version 20 (Released 2011; IBM Corp., Armonk, New York, USA). 

## Results

From 2016 to 2019, 4730 patients with PPCM were reported to the national inpatient sample database, 309 of whom developed VT (6.5%). Demographic data for these patients is summarized in Table 1 based on VT status. Age and primary expected payer were significantly associated with VT in PPCM, with VT patients being older on average than non-VT patients (34.6 ± 8.0 vs 31.9 ± 7.1, p=0.001) and increased prevalence of Medicare insurance among VT patients (69 (22.3%) vs 358 (8.1%), p=0.001).

**Table 1:** Baseline characteristics of the study population of peripartum cardiomyopathy patients stratified on the basis of developing VT vs not. The data has been represented as n (%) or mean ± SD. P values significant at <0.05. VT: ventricular tachyarrhythmia.

Variable	Non-ventricular tachycardia, N (%)	Ventricular tachycardia, N (%)	P-value
Age	31.9 ± 7.1	34.6 ± 8.0	0.001 (T value 5.773)
Primary expected payer			0.001
Medicare	358 (8.1%)	69 (22.3%)	
Medicaid	2338 (53.0%)	147 (47.6%)	
Private insurance	1469 (33.3%)	79 (25.6%)	
Self-pay	138 (3.1%)	7 (2.3%)	
No charge	6 (0.1%)	1 (0.3%)	
Other	105 (2.4%)	6 (1.9%)	
Race			0.068
White	1622 (37.9%)	101 (33.4%)	
Black	1876 (43.8%)	158 (52.3%)	
Hispanic	469 (11.0%)	30 (9.9%)	
Asian or Pacific Islander	122 (2.8%)	4 (1.3%)	
Native American	52 (1.2%)	2 (0.7%)	
Other	140 (3.3%)	7 (2.3%)	
Median household income national quartile for patient zip code			0.539
0-25th percentile	1879 (42.9%)	135 (44.4%)	
26th to 50th percentile	1109 (25.3%)	78 (25.7%)	
51st to 75th percentile	906 (20.7%)	53 (17.4%)	
76th to 100th percentile	484 (11.1%)	38 (12.5%)	

Table 2 summarizes data from univariate analysis of comorbidities present in PPCM patients with and without VT. PPCM patients who developed VT were found to have a higher prevalence of cardiac comorbidities such as atrial fibrillation and coronary artery disease. They were also found to have a higher burden of pulmonary conditions such as pulmonary hypertension and obstructive sleep apnea (OSA). Chronic kidney disease (CKD) was also more prevalent in the VT cohort. Conversely, the analysis showed a lower burden of obstetric conditions among PPCM patients with VT, including gestational diabetes, gestational hypertension, and pre-eclampsia. 

**Table 2:** Analysis of comorbidities of peripartum cardiomyopathy patients by ventricular tachycardia status. COPD: chronic obstructive pulmonary disease. The data has been represented as n (%). P values significant at <0.05.

Variable	Non-ventricular tachycardia, N (%)	Ventricular tachycardia, N (%)	P-value
COPD	744 (16.8%)	55 (17.8%)	0.660
Coagulopathy	127 (2.9%)	24 (7.8%)	0.001
Cardiovascular disease	22 (0.5%)	4 (1.3%)	0.067
Diabetes mellitus type II	424 (9.6%)	38 (12.3%)	0.121
Hypertension	317 (7.2%)	28 (9.1%)	0.216
Alcohol abuse	39 (0.9%)	4 (1.3%)	0.460
Liver disease	163 (3.7%)	16 (5.2%)	0.184
Peripheral vascular disease	4 (0.1%)	0 (0.0%)	0.597
Atrial fibrillation	188 (4.3%)	70 (22.7%)	0.001
Hypothyroidism	230 (5.2%)	23 (7.4%)	0.091
Coronary artery disease	124 (2.8%)	22 (7.1%)	0.001
Pulmonary hypertension	479 (10.8%)	63 (20.4%)	0.001
Tobacco use disorder	33 (0.7%)	0 (0.0%)	0.127
Obstructive sleep apnea	207 (4.7%)	42 (13.6%)	0.001
Iron deficiency anemia	560 (12.7%)	43 (13.9%)	0.524
Chronic kidney disease	326 (7.4%)	53 (17.2%)	0.001
Gestational diabetes	135 (3.1%)	3 (1.0%)	0.035
Gestational hypertension	197 (4.5%)	5 (1.6%)	0.017
Pre-eclampsia	671 (15.2%)	9 (2.9%)	0.001
Cocaine use	53 (1.2%)	6 (1.9%)	0.255
Opioid use disorder	96 (2.2%)	8 (2.6%)	0.628
Obesity	477 (10.8%)	43 (13.9%)	0.089

A second univariate analysis was conducted to study the outcomes of PPCM patients with and without VT, as shown in Table 3. Independent sample T-test analysis showed that the length of hospital stay and hospitalization costs were significantly longer in those who developed VT. Multiple outcomes varied significantly by VT status, such as mortality during hospitalization, with an increased mortality rate in the VT group vs the non-VT group (12 (3.9%) vs 55 (1.2%), p<0.001). Other outcomes that were more prevalent among the VT cohort include cardiogenic shock, vasopressor use, intra-aortic balloon pumps, and mechanical ventilation. 

**Table 3:** Analysis of outcomes of peripartum cardiomyopathy patients by ventricular tachycardia status. The data has been represented as n (%) or mean ± SD. STEMI: ST-elevation myocardial infarction; NSTEMI: non-ST-elevation myocardial infarction. P values significant at <0.05.

Variable	Non-ventricular tachycardia, N (%)	Ventricular tachycardia, N (%)	P-value
In-hospital mortality	55 (1.2%)	12 (3.9%)	0.001
Length of stay (days)	5.34 ± 3.5	13.11 ± 8	0.001 (T value=6.209)
Total charges ($)	76,318 + 3380	253,998 ± 28,500	0.001 (T value=6.178)
Permanent pacemaker	5 (0.1%)	0 (0.0%)	0.554
Cardiogenic shock	209 (4.7%)	68 (22.0%)	0.001
Vasopressor	36 (0.8%)	16 (5.2%)	0.001
Intra-aortic balloon pump (IABP)	56 (1.3%)	31 (10.0%)	0.001
Mechanical ventilation	134 (3.0%)	19 (6.1%)	0.003
Cardiac arrest	65 (1.5%)	23 (7.4%)	0.001
STEMI	5 (0.1%)	5 (1.6%)	0.001
NSTEMI	148 (3.3%)	14 (4.5%)	0.269

**Figure 1:**
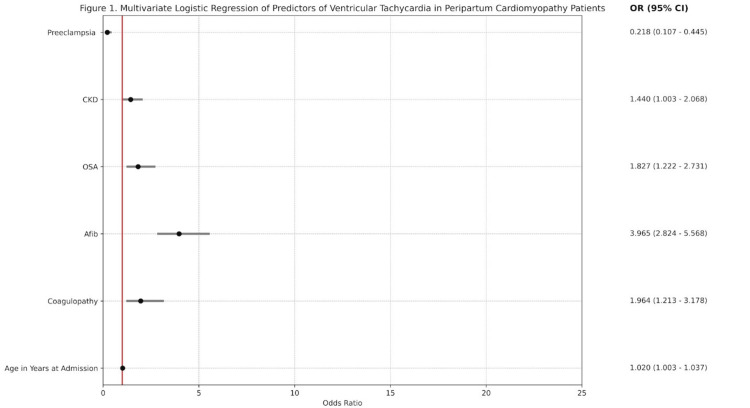
Multivariate logistic regression of predictors of ventricular tachycardia in peripartum cardiomyopathy patients. CKD: chronic kidney disease; OSA: obstructive sleep apnea; Afib: atrial fibrillation.

We conducted a multivariate logistic regression to evaluate the predictors of VT in PPCM, as summarized in Figure 1. Age in years at admission was a demographic predictor significantly associated with an increased risk of VT in PPCM patients (adjusted OR (aOR)=1.020, 95% CI 1.003-1.037, p=0.023). Additionally, comorbidities that were significantly associated with an increased risk of VT in PPCM patients were coagulopathy (aOR=1.964, 95% CI 1.213-3.178, p=0.006), atrial fibrillation (aOR=3.965, 95% CI 2.824-5.568, p=0.001), OSA (aOR=1.827, 95% CI 1.222-2.731, p=0.003), and CKD (aOR=1.440, 95% CI 1.003-2.068, p=0.048). Conversely, pre-eclampsia was significantly associated with a decreased risk of VT in PPCM patients (aOR=0.218, 95% CI 0.107-0.445, p=0.001).

**Figure 2:**
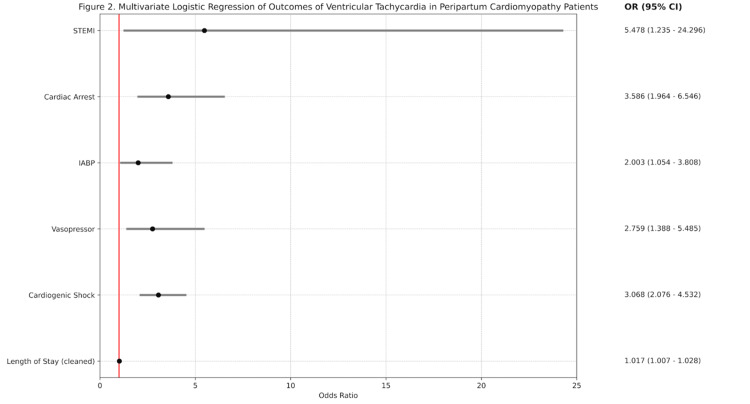
Multivariate logistic regression of outcomes of ventricular tachycardia in peripartum cardiomyopathy patients. STEMI: ST-elevation myocardial infarction, IABP: intra-aortic balloon pump.

We conducted a second multivariate logistic regression to determine the independent association of VT with outcomes in patients with PPCM, as summarized in Figure 2. Following adjustment for confounding variables, including age, race, and comorbidities, several outcomes were more likely in PPCM patients with VT. These outcomes included cardiogenic shock (aOR=3.068, 95% CI 2.076-4.532, p=0.001), vasopressor use (aOR=2.759, 95% CI 1.388-5.485, p=0.004), IABP (aOR=2.003, 95% CI 1.054-3.808, p=0.034), cardiac arrest (aOR=3.586, 95% CI 1.964-6.546, p=0.001), and STEMI (aOR=5.478, 95% CI 1.235-24.296, p=0.025).

## Discussion

In the present study examining VT in patients admitted with PPCM from 2016 to 2019, we found the prevalence to be 6.5%, warranting further analysis of VT’s predictors, comorbidities, and effect on outcomes. Among demographic variables, increased age was significantly associated with VT in PPCM. Advanced maternal age is an established risk factor for PPCM, with 50% of cases occurring in females above age 30 [5,12]. Furthermore, older age has been associated with increased mortality in a variety of conditions, including ventricular tachyarrhythmia [13]. Further studies are needed to investigate the impact of age on mortality among PPCM presenting with VT.

In the present study, multiple comorbidities were found to have significant associations with PPCM. On univariate analysis, VT patients were found to have a higher prevalence of atrial fibrillation (Afib). Vaidya et al. report Afib to be the most prevalent arrhythmia (27 per 100,000) among hospitalized pregnant females [14]. Recent research suggests that Afib is a risk factor for lethal arrhythmias, including VT [15]. This supports the results of our multivariate regression, which showed Afib to be an independent predictor of VT among PPCM patients. Recent research has also found Afib to be linked to increased mortality among pregnant women [16]. The prevalence and severity of outcomes associated with Afib call for further investigation into the role it plays in the development of VT in PPCM and its influence on outcomes in these patients.

Multivariable analysis showed that multiple comorbidities were significantly associated with VT in PPCM patients, among them coagulopathy, OSA, and CKD. The increased prevalence of OSA in VT patients found in this study is consistent with current literature, with one study showing OSA to be associated with increased odds of pregnancy-related cardiomyopathy. According to the aforementioned study, women with OSA experienced more than fivefold increased odds of in-hospital mortality [17]. With regard to the relationship between the cardiovascular and renal systems, a previous study showed CKD to be the strongest clinical risk factor for PPCM among women with pre-eclampsia. The study showed CKD to be independently associated with superimposed PPCM among women diagnosed with pre-eclampsia [18]. The observed associations with VT align with prior research linking OSA and CKD to adverse outcomes, underlining the need for intensified research efforts and more aggressive risk factor modifications in individuals with these comorbidities to clinically target these risk factors, improve prognoses and guide clinical interventions effectively.

Disorders of pregnancy, such as pre-eclampsia, gestational diabetes mellitus (GDM), and gestational hypertension, warrant a thorough investigation in the context of PPCM and VT. Our study reports a lesser prevalence of disorders of pregnancy in those who develop VT in PPCM as opposed to those who do not develop VT. While there is a paucity of data conveying the association between disorders of pregnancy and VT, many studies elucidate structural remodeling of the heart in GDM and pre-eclampsia [18,19]. The CARDIA trial analyzed echocardiograms recorded over a 20-year period in pregnant females. After adjusting for potential confounders, the paper reported that women with prior GDM had an increased left ventricular mass index, impaired relaxation, and systolic dysfunction by the end of the study [19]. In agreement with this, Aksu et al. report that left ventricular mass index and relative wall thickness were significantly higher in pre-eclampsia [20]. Although one would expect structural heart changes to be a nidus for the development of arrhythmias such as VT, our findings report otherwise. More research is needed on the association of cardiovascular disorders of pregnancy and fatal arrhythmias to determine how we can best utilize this information to improve patient outcomes.

Limitations of this study include that it is a retrospective study; therefore, causation cannot be inferred from the data analysis. Data were obtained from the National Inpatient Sample Database, an administrative database that uses ICD-10 codes and is thus prone to human coding errors. Another limitation is that our diagnosis of ventricular tachycardia was not classified as sustained vs non-sustained VT. 

## Conclusions

In this study, we found that multiple comorbidities are independently associated with ventricular tachycardia in patients with peripartum cardiomyopathy, including atrial fibrillation, CKD, and OSA. We also found that patients with peripartum cardiomyopathy who developed VT were less likely to have comorbid disorders of pregnancy, including gestational diabetes and gestational hypertension. The results of this study contribute to our current understanding of PPCM and its association with arrhythmias such as VT, thus enabling us to better recognize patients at risk, treat them more efficiently, and improve patient outcomes.

## References

[1] Bauersachs J, König T, van der Meer P (2019). Pathophysiology, diagnosis and management of peripartum cardiomyopathy: a position statement from the Heart Failure Association of the European Society of Cardiology Study Group on peripartum cardiomyopathy. Eur J Heart Fail.

[2] Rodriguez Ziccardi M, Siddique MS (2023). Peripartum cardiomyopathy. StatPearls (Internet).

[3] Bhattacharyya A, Basra SS, Sen P, Kar B (2012). Peripartum cardiomyopathy: a review. Tex Heart Inst J.

[4] Gammill HS, Chettier R, Brewer A, Roberts JM, Shree R, Tsigas E, Ward K (2018). Cardiomyopathy and preeclampsia. Circulation.

[5] Koenig T, Hilfiker-Kleiner D, Bauersachs J (2018). Peripartum cardiomyopathy. Herz.

[6] Cunningham FG, Byrne JJ, Nelson DB (2019). Peripartum cardiomyopathy. Obstet Gynecol.

[7] Ramlakhan KP, Johnson MR, Roos-Hesselink JW (2020). Pregnancy and cardiovascular disease. Nat Rev Cardiol.

[8] MacDorman MF, Declercq E, Cabral H, Morton C (2016). Recent increases in the U.S. maternal mortality rate: disentangling trends from measurement issues. Obstet Gynecol.

[9] Sliwa K, Förster O, Libhaber E, Fett JD, Sundstrom JB, Hilfiker-Kleiner D, Ansari AA (2006). Peripartum cardiomyopathy: inflammatory markers as predictors of outcome in 100 prospectively studied patients. Eur Heart J.

[10] Mallikethi-Reddy S, Akintoye E, Trehan N (2017). Burden of arrhythmias in peripartum cardiomyopathy: analysis of 9841 hospitalizations. Int J Cardiol.

[11] Goland S, Modi K, Bitar F (2009). Clinical profile and predictors of complications in peripartum cardiomyopathy. J Card Fail.

[12] Davis MB, Arany Z, McNamara DM, Goland S, Elkayam U (2020). Peripartum cardiomyopathy: JACC state-of-the-art review. J Am Coll Cardiol.

[13] Weidner K, Schupp T, Rusnak J (2023). Impact of age on the prognosis of patients with ventricular tachyarrhythmias and aborted cardiac arrest. Z Gerontol Geriatr.

[14] Vaidya VR, Arora S, Patel N (2017). Burden of arrhythmia in pregnancy. Circulation.

[15] Kim YG, Choi YY, Han KD (2021). Atrial fibrillation is associated with increased risk of lethal ventricular arrhythmias. Sci Rep.

[16] Lee MS, Chen W, Zhang Z (2016). Atrial fibrillation and atrial flutter in pregnant women-a population-based study. J Am Heart Assoc.

[17] Louis JM, Mogos MF, Salemi JL, Redline S, Salihu HM (2014). Obstructive sleep apnea and severe maternal-infant morbidity/mortality in the United States, 1998-2009. Sleep.

[18] Malhamé I, Dayan N, Moura CS, Samuel M, Vinet E, Pilote L (2019). Peripartum cardiomyopathy with co-incident preeclampsia: a cohort study of clinical risk factors and outcomes among commercially insured women. Pregnancy Hypertens.

[19] Appiah D, Schreiner PJ, Gunderson EP (2016). Association of gestational diabetes mellitus with left ventricular structure and function: the CARDIA Study. Diabetes Care.

[20] Aksu E, Cuglan B, Tok A, Celik E, Doganer A, Sokmen A, Sokmen G (2022). Cardiac electrical and structural alterations in preeclampsia. J Matern Fetal Neonatal Med.

